# Herringbone micromixers for particle filtration

**DOI:** 10.1063/5.0134431

**Published:** 2023-01-23

**Authors:** Jacob L. Binsley, Thomas O. Myers, Stefano Pagliara, Feodor Y. Ogrin

**Affiliations:** 1 Department of Physics and Astronomy, University of Exeter, Exeter EX4 4QL, United Kingdom; 2 Platform Kinetics Limited, Pegholme, Wharfebank Mills, Otley LS21 3JP, United Kingdom; 3 University of Exeter, Living Systems Institute and Biosciences, Exeter EX4 4QD, United Kingdom

## Abstract

Herringbone micromixers are a powerful tool for introducing advection into microfluidic systems. While these mixers are typically used for mixing fluids faster than the rate of diffusion, there has been recent interest in using the device to enhance interactions between suspended particles and channel walls. We show how the common approximations applied to herringbone micromixer theory can have a significant impact on results. We show that the inclusion of gravity can greatly alter the interaction probability between suspended particles and channel walls. We also investigate the proposed impedance matching condition and the inclusion of imperfect binding using numerical methods, and investigate transient behaviors using an experimental system. These results indicate that while traditional methods, such as simple streamline analysis, remain powerful tools, it should not be considered predictive in the general case.

## I. INTRODUCTION

Herringbone micromixers (HBMs), first developed in 2002,^1^ have seen much use as an effective method to introduce advection into laminar flow devices in microfluidics. Advection is pivotal for mixing fluids at rates faster than the limit of diffusion,^1,2^ while simultaneously narrowing residence times.^3^ This mixing enhancement has led to HBMs being explored in the interest of a number of novel applications, from diagnostics^4,5^ to targeted medicine.^6–8^

The behavior of herringbone micromixers has been explored in the context of low Reynolds number^9^ mixing both experimentally and numerically.^2–13^ This surfeit of research has garnered an expansive understanding of certain key aspects of the behavior of HBMs, such as the sensitivity of performance to changes in key variables,^9^ and has allowed for the generation of simple models capable of extracting key optimization criteria for various applications.

One suggestion with particularly interesting implications is the use of HBMs to enhance the probability of interactions between suspended particles and the walls of the mixing device in use.^4–14^ These interactions are of particular importance when considering enhanced detection in medical devices,^5^ and as such, surface interaction probability is the metric used in this paper.

A novel avenue of investigation is the use of HBMs as an integrated filtration device within point-of-care (POC) systems. Lab-on-a-chip (LOC) microfluidic devices rely on miniaturized versions of laboratory processes, allowing for the manipulation of small volumes of fluid. The repertoire of miniaturized components is ever growing, encompassing a range of capabilities from mixing^15^ and signaling,^16^ to integrated pumping mechanisms.^17–19^ While some miniaturized filtration systems have been developed for this scale,^20–22^ we still rely on macroscopic techniques within commercial applications.^23^ Bringing these filtration methods onto the chips themselves is an important step in allowing for samples of whole blood to be introduced to POC systems where only the blood plasma is to be analyzed. This integration allows usage of the system without being preceded by a full lab-scale filtration process such as centrifuging. Therefore, in continuing the hunt for novel filtration components for POC systems, we are exploring the use of HBMs.

Typical numerical studies of HBMs do not move beyond the use of streamlines or fluid packets.^24–26^ These techniques assume that a particles motion is fully described by the fluid flow which carries it and does not allow for the inclusion of additional particle forces such as gravity. As such, there is a gap in the search for validity with the current choice of numerical techniques. Numerical validity is particularly poignant for studies contingent on evaluating particle–wall interactions^10^ where the primary focus is no longer on the relative motion of streamlines far from boundaries, but instead is exposed to the myriad perturbations associated with the flow profile of a particle moving relative to a nearby surface, subject to forces such as gravity or lift.

When including particle–fluid interactions, the simulations must become time-dependent to evaluate the positions of particles at each moment in time. The computational expense is therefore greatly increased compared to equivalent streamline models. Given the extent of this increased expense, it is reasonable that the fluid–particle interactions are typically eschewed if they are not assumed to significantly alter the expected outcome. However, given recent improvements in the capabilities of computer hardware and software, we are now able to perform expensive computations on a scale which has not before been feasible. As such, this study aims to shed light on the validity of the common assumption that acceleration due to gravity is negligible when simulating HBMs by performing the expensive calculations necessary to delve deeper into the particle–wall interactions.

Another key metric that is routinely overlooked when considering surface interaction between particles and channel walls is how this interaction rate may change over time in an experimental system: when the binding affinity between the surface and the particle are high, the particle will bind irreversibly, permanently occupying a potential binding site and theoretically reduce the performance of the device. This change in performance may be a concern when using high particle concentrations for prolonged durations. Therefore, we also compare the performance to transient experimental results, not only to verify simulation, but to further characterize the device.

This study aims to explore the dependence of an effect which cannot be easily explored with streamlines alone: the inclusion of gravity acting on non-neutrally buoyant particles. This force is understandably dismissed in typical investigations given that the associated perturbations of velocity for typical particles in a representative HBM are orders of magnitude smaller than the velocity in the driven flow direction. However, it is hypothesized that this small force is not entirely negligible. Even when no interaction is observed, particles are routinely circulated in close vicinity to the walls of the HBM. Therefore, even a small correction to the particle’s position may be the difference between detecting an interaction event or not.

## II. METHODS

### A. Numerical model

**FIG. 1.**
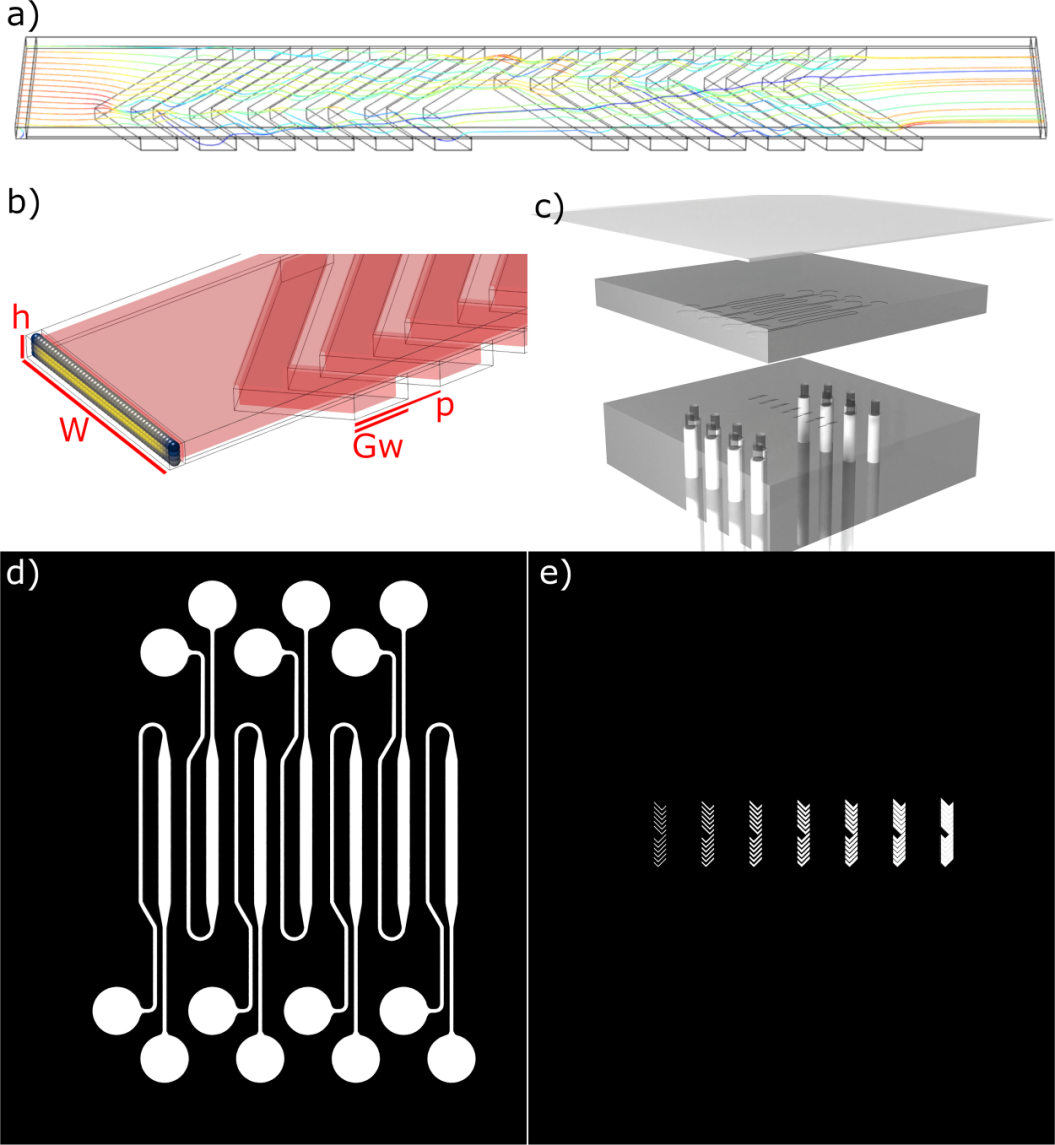
(a) Geometry of the design, showing γ=2.5. The outer boundary indicates the boundaries for fluid simulation. The streamlines indicate fluid flow from the inlet (left) to the outlet (right). (b) A close up of the inlet geometry and initial particle positions in a strict grid pattern. The color of the particles represents their initial velocity, with the yellow particles in the center of the channel beginning with peak velocity, and the blue particles in the corners beginning with minimum velocity. The transparent outer boundary indicates the boundary for fluid simulations as before, while the red inner boundary represents the boundary for particle simulations as addressed in the text. (c) The experimental device consists of two layers of PDMS, the top layer contains the geometry for the main fluid channel, while the bottom layer contains the geometry of the grooves. A glass slide is affixed to the top, and PTFE tubing is attached underneath to act as inlet and outlet, leading from a syringe pump. (d) and (e) Example photomask designs used in these experiments. Black represents emulsion to block UV-light, and white represents clear acetate which allows through UV-light to expose the relevant region. (d) shows the geometry of the channels. (e) shows the herringbone grooves.

A single staggered HBM cycle was modeled using COMSOL Multiphysics 5.6 [Fig. 1(a)], consisting of six grooves per half cycle, describing a rectangular cross section channel with 90 ${}^{{}^{\circ}}$ chevron shaped grooves carved into the bottom surface. The leading corner of each chevron is positioned one third the distance from the channel wall, meeting at a 45 ${}^{{}^{\circ}}$ angle with the mentioned wall. This design is mirrored on the opposite side after one half cycle in the typical fashion of a staggered HBM. The pitch between the two half cycles is kept constant such that the groove spacing along one side of the mixer is continuous. The height of the channel, h, is 50 μm, the width of the channel, w, is 500 μm, the depth of the grooves, d, is 50 μm (the same as h) and the pitch of the grooves, p is 200 μm. These metrics are labeled in Fig. 1(b). This particular geometry is intended to closely match a previous numerical study^10^ relying on streamline analysis, which will enable a point of reference when analyzing these results. The groove width, $G_{w}$ is varied as the independent geometric variable, and groove widths are represented here as a unitless ratio, γ, normalized against the channel height.

The fluid used is water, with density 1 g cm^ −3^ and dynamic viscosity 1 mPa s, subjected to the Navier–Stokes and continuity equations for laminar flow, and is discretized quadratically with respect to velocity and linearly with respect to pressure. The choice of discretization allows for reduced numerical diffusion^27^ while allowing for a reasonable mesh density, without unreasonable computational expense. Simulations have typical degrees of freedom on the order of 10^6^.

The outer walls impose a no-slip boundary condition and define the limits of the fluid simulations. However, COMSOL considers particles as point objects, and so to ensure realistic interaction behavior, a standoff boundary is defined one particle radius within the outer wall [Fig. 1(b)] such that when a point particle interacts with this boundary; the outer radius of that particle simultaneously interacts with the outer wall. This inner boundary is not considered in fluid flow calculations aside from altering the shape of the mesh elements.

An inlet region with a different recession from the outer walls is also defined as part of this inner boundary. This inner boundary is for ease of introduction of particles, allowing for the definition of a square boundary mesh whereby all of the desired particle starting locations can be assigned to the center of each mesh element. The size of these mesh elements is of equivalent size as the other elements and does not reduce the accuracy of the flow simulations. The change in shape of the inner boundary is inconsequential as it does not result in any particle interactions in this region, since all particles follow approximately straight trajectories at the inlet. Therefore, the added convenience is not seen to alter the solution in any meaningful way.

Fluid flow is calculated using a stationary solver. Solving the Navier–Stokes equation and assuming a slip boundary condition. 196 particles are added at the inlet at equally spaced coordinates, 10 μm apart, with a 10 μm depletion region around the edge to avoid particles immediately interacting with the walls *ab initio*. The depletion region is not only prudent given the nature of depletion regions near boundaries in straight channels, but also replicates the inlet conditions of a previous reference paper.^10^ Particle–wall interactions are counted as contributing to specific interaction surfaces: for example, segregating interactions with the channel top from interactions with the channel bottom. Breaking down the interactions in this way can help us understand some of the trends exhibited by the system.

The particles are simulated in a one-way coupled manner, with the particles experiencing the effects of the fluid, but without altering the path of the flow in response. The particles cannot interact with each other, nor impact the flow in any way. This condition represents a physical system in the limit of particles with a low number density. The particle trajectories are calculated in a time dependent manner with a maximum step size, $\tau =P/1000\cdot \bar{v}$, where p is the pitch and $\bar{v}$ is the mean inlet velocity. The simulations continue until 95% of the particles have interacted with the walls or the outlet. The simulations are stopped early in this manner due to diminishing returns toward the increase in accuracy against the expense of the computation and is consistent with previous studies on similar systems which found that as many as 3% of particles can become trapped following closed loops;^24^ it is unreasonable to expect the paths of all inlet particles to be fully resolved in a reasonable time frame in the general case.

Particles enter with velocities defined by fully developed inlet flow and experience Stokes drag which carries the particles with the fluid. The flow profile at the inlet is chosen to be fully developed rather than constant in order to realistically match the physical system. Since we are simulating particles, not streamlines, we are free to add contributions due to gravity. Acceleration due to gravity is added through the inclusion of the Archimedes principle. Simulation outputs were consolidated using Matlab livelink.

### B. Experimental fabrication and analysis

The devices were fabricated using SU-8 monolithography. A photomask was designed in LibreCAD and printed on an emulsion film by JD Photodata. The photomask contains two layers [Figs. 1(d) and 1(e)], showing both the main channels and the grooves on separate layers. The masks define multiple channels, each aligned with grooves of various widths.

To fabricate, a HMDS based primer was spin coated onto a 2.5 cm square silicon wafer at 4000 rpm and baked at 95 ${}^{{}^{\circ}}$C for 10 min. These wafers were then spin coated with SU-8 3050 at 1500 rpm resulting in a layer thickness of 50 μm.

The wafers were then baked at 95 ${}^{{}^{\circ}}$C for 45 min and then exposed to UV light in a Kloe Kube through the photomask for 6 s at 95% power. The wafer could then be baked at 65 ${}^{{}^{\circ}}$C for 2 min and 95 ${}^{{}^{\circ}}$C for 3 min. Finally, the wafer is washed in a PGMEA based developer until the uncured SU-8 was removed, leaving only the completed molds.

These molds were then placed into 3D printed holders and covered in PDMS (mixed 10:1 oligomer to curing agent) and baked at 100 ${}^{{}^{\circ}}$C for 15 min until cured. The two layers were then combined with the “stamp and stick” method,^28^ by spin coating a 2.4 cm glass slide with PDMS at 7000 rpm.

The resulting device (Fig. 1) consists of two PDMS layers defining the two layers of the geometry, a glass slide, and inlet/outlet tubing connected to a syringe pump.

A syringe pump was then used to pump a test solution consisting of 2:9 glycerol:water, and 2400:1 fluid to 15 μm diameter, spherical polystyrene beads (w/w); at a rate of 0.1 ml/h ensuring a Reynolds number of ∼0.1 through the micromixer. The resultant fluid density is 1.05 g cm^ −3^ (matching that of the polystyrene beads resulting in a neutrally buoyant suspension) and the dynamic viscosity is 1.62 mPa s. This medium is not equivalent to applications using whole blood, but is able to offer a point of comparison to the model used, and so is an important verification tool. The use of such inert polystyrene beads as a substitute for biological materials when considering physicochemical interactions is not unusual, although it is important to note that results may vary significantly based on surface chemistry.

The first half-cycle of the grooves was observed through a 10 × optical microscope connected to a camera recording at 10 frames per second for 6545 frames (or 654.5 s). Over this time period, beads are observed to settle within the device. A particle is considered captured if it remains stationary for the remainder of the experiment. The number of beads captured in the region of interest is measured as a function of time and fit to a curve (example given in Fig. 5 inset).

## III. RESULTS AND DISCUSSION

### A. Simulation

**FIG. 2.**
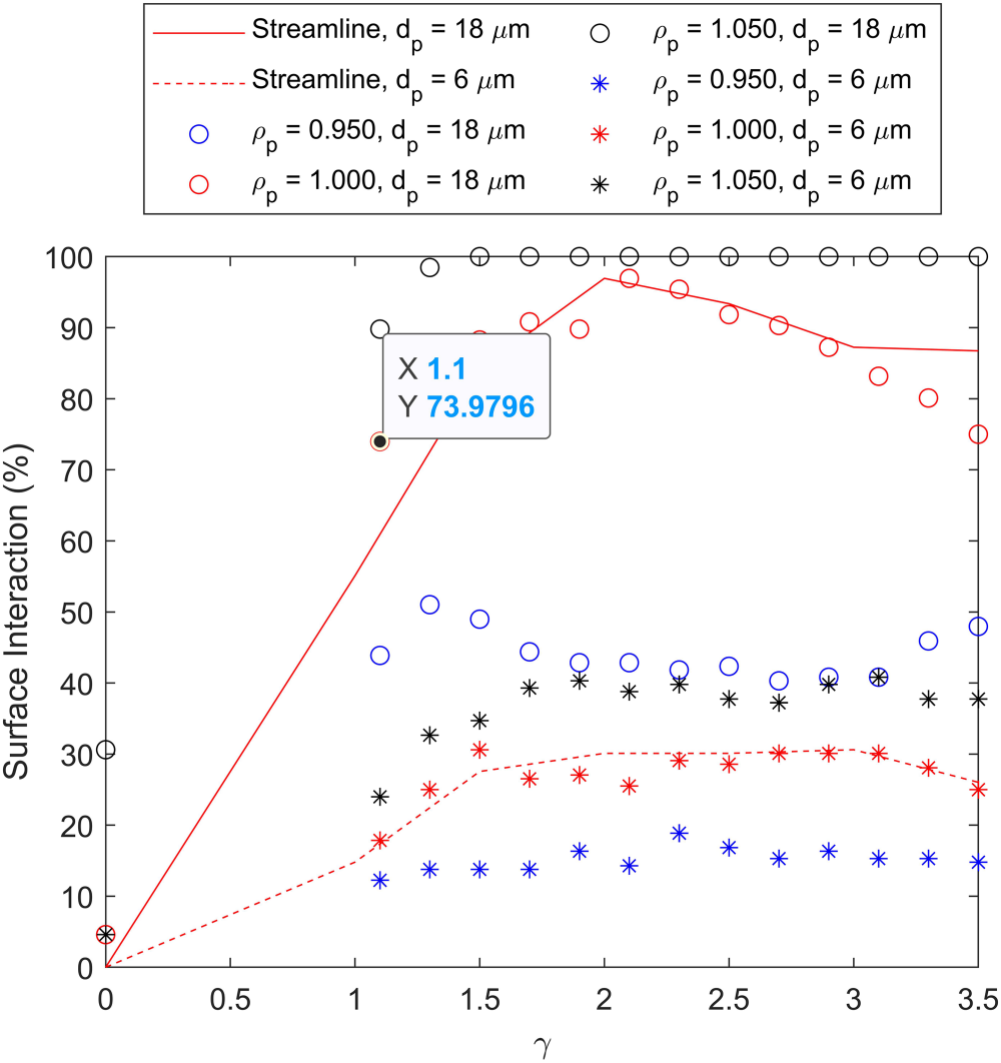
Simulating the probability of a particle interacting with the wall of the micromixer as a function of γ. Streamline simulations of varying particle diameter, $d_{p}$ are represented by lines. Particle simulations of varying $d_{p}$ and particle density, $\rho_{p}$ (normalized against the density of the fluid) are represented as discrete points.

We start by performing a streamline analysis of an HBM to verify our model against previous work (Fig. 2). We find similar trends in surface interaction as a function of γ to previously published work.^1^ A peak interaction rate of 97% occurs at γ=2.0 for particles with a diameter of 18 μm. When the particle diameter is reduced, the peak becomes much shallower and broader since the particles must get closer to the walls before interacting.

We then repeat the simulations using fluid–particle interactions rather than assuming the motion of particles using streamlines (Fig. 2), allowing us to include the effects of gravity. Neutrally buoyant particles closely follow streamlines, as is expected when the forces on the particle are dominated by drag alone; carrying it with the fluid. In the absence of acceleration due to gravity, in a low Reynolds number regime where inertial effects can be ignored, the results show agreement with the curves represented in previous streamline studies.

Particles that are denser than the surrounding fluid experience a net downward gravitational force, altering their positions within the channel over time to stray from that defined by the streamlines. When the particles are 5% denser than the fluid, such as might be expected from polystyrene tracer beads in water, the capture rate increases compared to the neutrally buoyant case. When particles are 5% less dense than the surrounding fluid, the interaction probability is reduced and becomes more constant with varying groove width. This behavior is because the gravitational force biases the particles away from the grooves, toward the top of the channel. It is important to note that at γ=0, a straight channel, the interaction probability is the same when particles are either more or less dense than the fluid, since it is the same system being mirrored.

The change in interaction probability as a function of particle density, $\rho_{p}$, is significant despite retaining a low sedimentation velocity. This behavior is enforced when considering particles of different diameters (Fig. 2). A particle with reduced diameter must be brought closer to the wall in order to interact, and thus, reduces the interaction probability.

Testing a particle diameter of 6 μm is significant since it is the approximate size of a spherical equivalent human red blood cell (RBC). If we assume a RBC has a volume of 90 fl,^29^ then simply approximating its shape to a sphere, gives us a diameter of 5.56 μm. This diameter is inline with other studies which approximate RBC’s as spheres using different methodologies.^30–32^ If a RBC has a density of 1.110 g m^ −333^ and its surrounding blood plasma has a density of 1.080 g cm^ −3^:^34^ the RBC is 4.3% more dense than the surrounding plasma. Therefore, our simulation results can be shown to give an estimate response for RBC sedimentation within a HBM (Fig. 2, black stars), with potential interest for applications in blood filtration for on-chip sample preparation in POC devices.

**FIG. 3.**
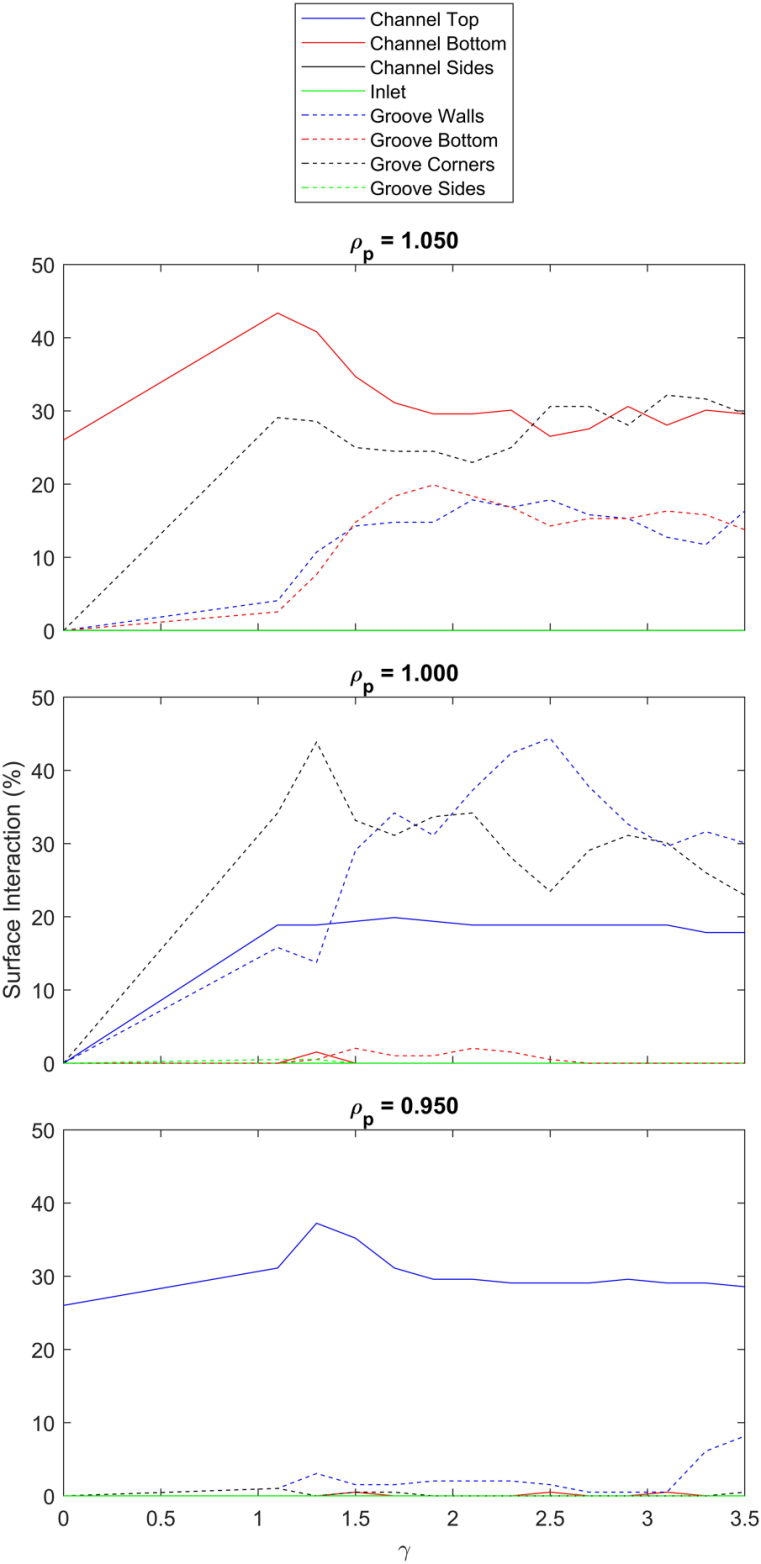
Simulating the interaction probabilities by surface for particles with diameter of 18 μm. Each plot represents a different normalized particle density, $\rho_{p}$ (normalized against the density of the fluid). For the neutrally buoyant case, $\rho_{p}=1.000$, the favored interaction surfaces lie at the interface between the grooves and the channel. Denser particles increase the interaction probability at the bottom of the channel and the bottom of the grooves due to the increased sedimentation velocity. Less dense particles see a reduction in interaction probability at the channel bottom in favor of an increased interaction probability with the channel top.

The change in interaction rate as a function of particle density can be better understood when considering the interaction surfaces involved (Fig. 3). It can be seen that the majority of interactions in the neutrally buoyant case take place in the vicinity of the interface between the grooves and channel. The interaction surface explains the dependence of interaction probability with groove width, since an impedance matching condition between flow in the grooves and flow in the channel increases the residency time of particles in this region.^2^ As explained eloquently by Hassel *et al.*, if the resistance of the groove is too high (the groove is too narrow) the groove will insufficiently perturb flow in the channel, and if the resistance of the groove is too low (the groove is too wide), the particles also remain in bulk fluid, only in this case favoring the grooves rather than the channel.

However, we find that the inclusion of gravity alters the favored interaction surfaces. Particles which are denser than the surrounding fluid sediment over time and increase the interaction probability with the channel bottom and groove bottom. Particles which are less dense than the surrounding fluid, experience a force away from the favored surfaces and toward the channel top. Therefore in this instance, the impedance matching condition is no longer a good predictor of peak interaction probability.

**FIG. 4.**
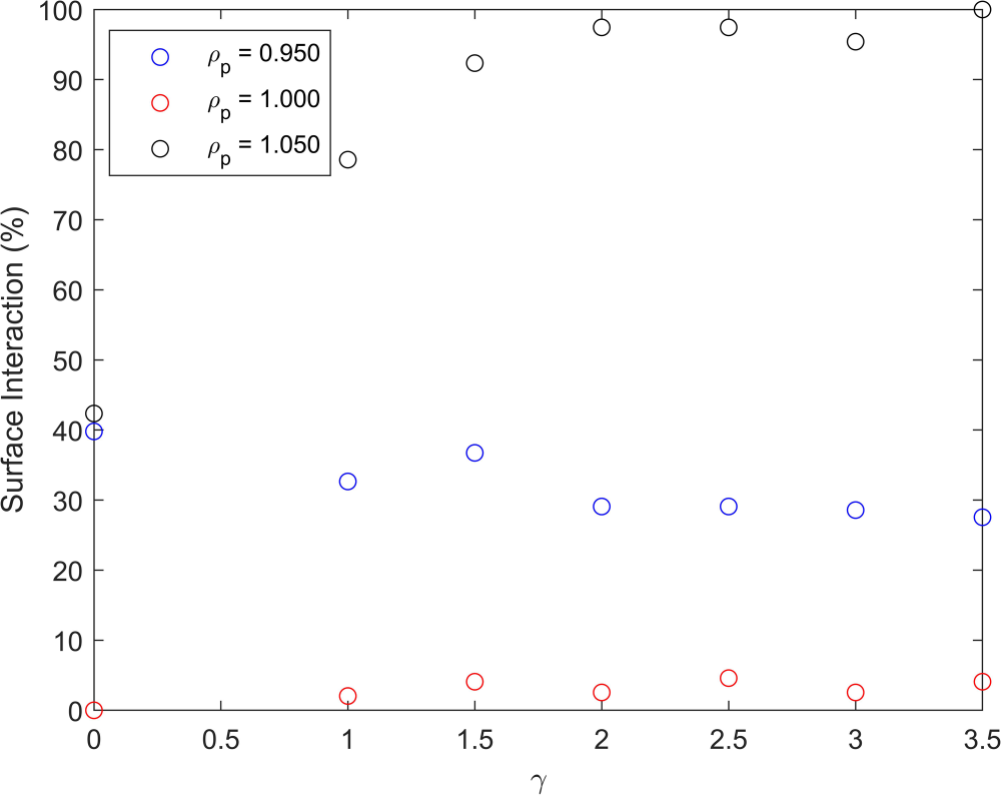
Simulating the interaction probabilities with a finite binding probability between particle and wall of 0.5%. Each curve represents a different normalized particle density, $\rho_{p}$ (normalized against the density of the fluid), as a function of γ.

These simulations show only the case of perfect binding between particle and wall, but we also consider the case of imperfect binding, modeled by the inclusion of a probability associated with the interaction event, resulting in the particle bouncing instead of binding (Fig. 4). The probability of an interaction event resulting in binding is 0.5%. This probability is not chosen based on any direct comparison to physical systems. Considering this imperfect binding shows a significant reduction in interaction rate for neutrally buoyant particles, as might be expected, although a much less significant change for non-neutrally buoyant particles. This robustness against imperfect binding is because the force of gravity acts as a bias to keep particles close to walls even after a failed capture event. Therefore, the binding affinity becomes less relevant, since the particle will sediment regardless.

**FIG. 5.**
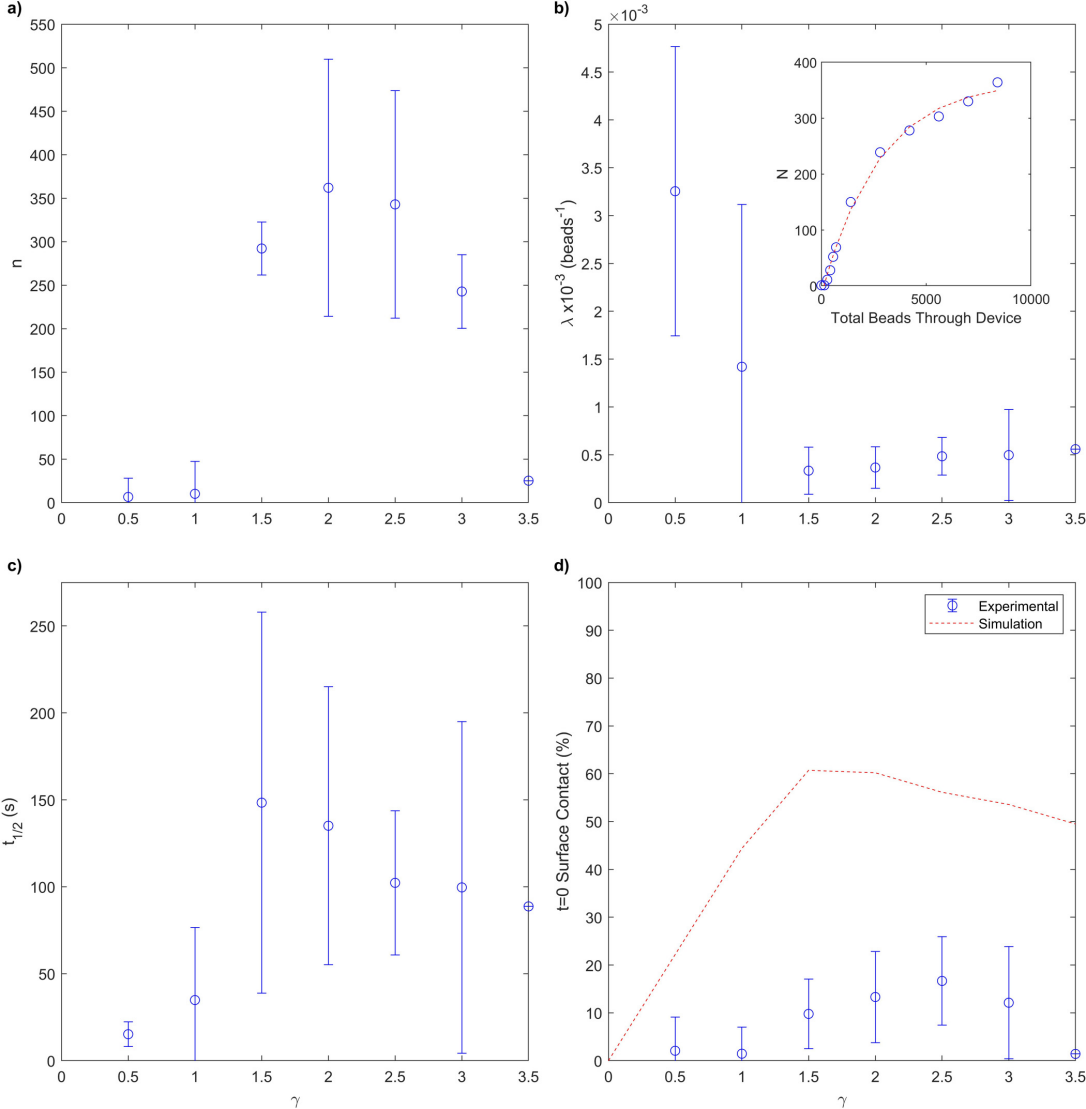
Experimental investigation of particle capture. (a)–(d), respectively represent as a function of γ the maximum capture capacity, decay constant (bead^ −1^), half-life and *ab initio* capture rate. The inset shows an example of the curve fitting to experimental data, measuring the number of beads sedimenting within the device over time; the example case is given for a measurement at a γ=2. (d) includes both experiment and simulation results. The simulations used for comparison show 15 μm diameter beads, a $\rho_{p}$ of 1, being neutrally buoyant with fluid and particle density of 1.05 g cm^ −3^, and only account for the particles captured by the first half cycle of the herringbone, corresponding to the field of view provided by our experimental system.

When replicating this system experimentally, the number of beads captured within a device over time is fitted to the function

$$N=n(1-e^{-\lambda (b-c)}),$$ (1)

where N is the number of particles captured, n is the maximum number of particles which the device is capable of capturing, λ is a decay constant, not relative to the passage of time, but relative to the number of beads which have passed through the device. b is the number of beads which have passed through the device. c is an offset based on time to account for any uncertainty at the start point of a measurement. This fit is obtained for γ in a range between 0.5 and 3.5 and an example fit is given in Fig. 5 inset. We find a peak value of n and a minimum value of λ at γ=2 and 1.5, respectively (Fig. 5). From our fitting function, we can calculate the theoretical capture rate at time, t=0 as with simulation [Fig. 5(d)]. We see a broad peak in interaction probability at γ=2.5.

Comparing this figure to what we expect from simulation [Fig. 5(d)], we find some agreement and some disagreement. Both indicate a broad peak capture rate of similar magnitude at intermediate values of γ, while a straight groove has a capture rate of 0. Experimental measurements are suppressed at γ=3.5 due to limited fabrication tolerances resulting in the grooves merging into one large groove, approximating a simple section of straight channel with double the value of h. We also find that the capture rate measured in experiment is significantly reduced compared to simulation. This reduction in capture rate is yet unknown, but could be for a number of likely reasons, such as the elimination of a given interaction surface (Fig. 3), or a finite binding affinity between particles and wall (Fig. 4).

The experimental data supporting Fig. 5 are obtained from the median performance across seven devices. Error bars are not calculated due to the limited number of repeats, although the data are available at the University of Exeter Repository.^35^

## IV. CONCLUSION

This paper agrees with typical streamline approximations in the case of neutrally buoyant particles as is to be expected due to the absence of additional forces. Allowing information of peak flow to be obtained from the analytical expressions explored in previous studies.

However, the simulations in this paper lead us to conclude that the effects of gravity cannot be ignored in cases where the particles are not neutrally buoyant. Even a deviation in particle density of 5% is enough to measurably alter the interaction probability. When particles are more or less dense than the surrounding fluid, the approximations break down and simple methods are not able to reasonably approximate the trend of surface interactions as a function of groove width.

When an equivalent system to RBCs in plasma is simulated (Fig. 2, black stars); the rate of surface interaction (and potential for cell capture) shows deviation from what would be predicted in a simple streamline simulation. In this case, since RBCs are denser than plasma, the probability of interaction is increased; improving the predicted viability of using HBMs for cell filtration and therefore sample preparation in POCs.

The experiments performed in this paper show partial agreement with simulations for time t = 0. The agreement allows for confidence when using simulations for the purpose of rapid prototyping, enabling faster design implementation for real-world applications when compared to iterating through experimental cycles. The experiments also highlight that the performance of the HBM decays rapidly over time as the available interaction sites become filled. The interaction is not captured in simulation since inter-particle interactions are not considered. This performance decay is more pronounced here since the devices consist of only a single HBM cycle, and may be mitigated by incorporating a sequence of grooves, totaling more available binding sites, increasing n. The reduced performance of the experimental system in comparison to the simulated one could also be attributed to by imperfect surface adhesion, as simulated in Fig. 4. If this is a contributing factor, then the reduced performance could be mitigated using non-neutrally buoyant particles, or by using surface coatings which improve adhesion; although testing this is beyond the current capability of our equipment.

This paper seeks to enlighten readers to the understanding that calculations involving this system are not accurate in the general case when ignoring gravitational effects. This is not a criticism of the standard methods, but provides supplementary understanding, especially given that these full particle–fluid interactions are far more computationally expensive than simple streamline models.

## Data Availability

The research data supporting this publication are openly available from the University of Exeter’s institutional repository at https://doi.org/10.24378/exe.4445.^35^
